# Properties of Journal Bearing Materials That Determine Their Wear Resistance on the Example of Aluminum-Based Alloys

**DOI:** 10.3390/ma14030535

**Published:** 2021-01-22

**Authors:** Alexander Mironov, Iosif Gershman, Eugeniy Gershman, Pavel Podrabinnik, Ekaterina Kuznetsova, Pavel Peretyagin, Nikita Peretyagin

**Affiliations:** 1 Laboratory of Electric Currents Assisted Sintering Technologies, Moscow State University of Technology “STANKIN”, Vadkovsky Lane 3a, 127055 Moscow, Russia; lecast.stankin@yandex.ru (A.M.); gershmanei@gmail.com (E.G.); p.podrabinnik@stankin.ru (P.P.); evkuznetsova11@gmail.com (E.K.); p.peretyagin@stankin.ru (P.P.); n.peretyagin@stankin.ru (N.P.); 2 Department of Scientific Research Programs, Grants and Projects, Railway Research Institute JSC “VNIIZHT”, 3rd Mytischinskaya Street 10, 107996 Moscow, Russia

**Keywords:** antifriction alloys, wear rate, seizure load, alloys composition, secondary structures, mechanical properties, third body, tribolayer

## Abstract

Potential relations of tribological characteristics of aluminum antifriction alloys with their compositions and mechanical properties were investigated. In this regard, the properties of eight aluminum alloys containing tin from 5.4% to 11% doped with lead, copper, silicon, zinc, magnesium, and titanium were studied. Mechanical properties such as hardness, strength, relative extension, and impact strength were analyzed. Within the tribological tests seizure load and wear of material were evaluated and secondary structures were studied afterwards. The absence of a definitive correlation between tribological behavior and mechanical properties was shown. It was determined that doping tin over 6% is excessive. The seizure load of the alloys increases with the magnesium content. Secondary structures of the alloys with higher wear rates contain one order less magnesium and tin.

## 1. Introduction

Antifriction alloys with a low coefficient of friction (COF) are widely used, for instance, in journal bearing and are in high demand. In turn, establishing relationships between tribological behavior and properties and the composition of rubbing materials is one of the main tasks of tribology. Typically, mechanical properties, e.g., tensile strength, yield stress, ductility, and hardness are considered as a criterion for the tribological characteristics of materials. The hardness parameter is considered to be the most frequently used one to describe the influence on tribological properties [1]. Particularly, in [2,3,4,5] the wear rate of titanium, antifriction aluminum alloy, and others is described as a function of material hardness. Therefore, wear reduction is related to hardening. In [6,7] reduction of the wear rate of steel powder sintered with boron coupled with coated corrosion-resistant steel is elucidated by the increase of hardness.

In [8], antifriction aluminum-based alloys were studied. It was noted that the addition of low-melting components such as Bi, Cd, In and Pb to the Al-Si-Cu system leads to an increase in hardness and reduction in wear rate. The simultaneous improvement of mechanical properties (hardness of microhardness) and wear rate was attested in [9,10].

However, there are enough counterexamples demonstrating materials with reduced hardness have a lower wear rate. For instance, while examining AlTiN-DLC (diamond-like carbon, DLC) coating [11] it was concluded that softer coating exhibits a lower wear rate due to carbon formation on the friction surface. In [12], wear and hardness reduction of superhard coatings based on boron carbides and nitrides appeared after the addition of graphite and molybdenum disulfide. In [13], the authors revealed a decreae of the wear in Fe-Ti-SiC based nanocomposite coating containing MgC2 due to graphite release which affected hardness. Paper [14] has documented decreasing in the wear rate of polyethylene by 1.2–1.5 times by adding complex compounds of Nd and Al while its hardness decreased by 1.2 times. In [15], different types of steel with the same hardness yielded different wear rates which confirms the absence of a definitive correlation between these two parameters. The authors attempted to link the wear rate with yield strength and microstructure. Being coated with crystalline and amorphous coatings, steel showed better performance in the latter case despite harder crystalline coating [16]. Applying polymers to a ceramic coating reduced both the wear rate and hardness [17].

Based on these studies it can be concluded that incrementing mechanical properties and, particularly, hardness, might cause either decreasing or reduction of wear rate. Therefore, these parameters cannot be considered as wear resistance criteria. It is worth mentioning that often reduction of wear with a contemporary decrease in hardness is caused by physicochemical reactions in material, e.g., graphite formation.

Several works are focused on studying the influence of varying additives to rubbing materials or lubricants on tribological properties. In [18], the effect of modifying rubber with nanocrystalline cellulose and graphene on tribological characteristics was studied. It is noted that the modified rubber has a reduced wear rate. In [19], the wear rate of the WC-Al_2_O_3_ composite at 600 °C was investigated. It is shown that the introduction of nickel into the material inhibits the decarburization of WC, increasing the wear rate. In [20], the wear of an aluminum wire rubbing with a disk with an anodized surface or a diamond-like coating was studied. It was demonstrated that the DLC reduces the wear rate more effectively. In [21], tribological tests of brake friction material with carbon fibers were carried out. It is shown that the introduction of carbon fibers leads to a decrease in the coefficient of friction and the intensity of wear. The addition of graphene and nanoparticles of molybdenum and tungsten to the oil resulted in an apparent decrease in the coefficient of friction and the intensity of wear [22]. Work [23] describes increasing in wear rate preceded by graphite release on the friction surface. However, the appearance of graphite on the friction surface does not necessarily lead to a decrease in the wear rate.

Lubricant properties are vital for mixed or fluid friction. Rubbed in different lubricants, materials with the same mechanical properties show different wear rates [24]. The friction coefficient is influenced by the wetting angle [25]. Environment and oxidation processes are responsible for wear mechanisms, as suggested in [26] while studying Ag-Al coatings. Investigation of DLC coatings in different lubricants with molybdenum additives revealed that the wear rate increases with an increase in the hydrogen content in the coating [27]. Tests of steel wire in different environments [28] showed that the oxidation rate of the wire does not correlate with the wear rate.

It has previously been observed that tribological properties are substantially affected by tribofilms formed during friction. Studying their formation mechanisms revealed the presence of two minima on the curves of the dependence of the coefficient of friction and the wear rate on the load [29]. This made it possible to expand the range of loads, for example, for worm gears. Surveys such as that conducted in [30] have shown that there is a deformed layer on the friction surface of titanium alloys. Interestingly, the wear rate was observed to reduce as twins emerge in the deformed layer. These relationships may be explained by self-organization during friction when the friction energy is spent on non-spontaneous processes not associated with friction [31]. Deformation is induced by friction; the formation of twins consumes part of the friction energy, which, without self-organization, could be spent on wear. In [32], a synergistic effect was found when studying the wear of the piston ring of a cylinder liner with a Ni-P-TiN coating while working in the oil contained Mo_2_S nanocomposite additive. The synergistic effect is expressed in a significant decrease in the wear rate. Thus far, previous studies have reported an increase in the wear rate with a simultaneous decrease in the coefficient of friction during the friction of synthetic diamonds in nitrogen [33]. The authors researched different environments [33]. The results obtained may be due to the effect opposite to synergistic [23,34]. Investigating the tribological behavior of the Si_3_N_4_-hBN ceramic composite showed the beneficial influence of seawater on the coefficient of friction and wear rate compared to deionized water. Friction in seawater is accompanied by tribolayer growth on the friction surface [35]. Furthermore, according to [36], a similar synergistic effect of reducing the friction coefficient and wear may occur place due to the combined action of (Ni_3_Al-Ag) and Al_2_O_3_ hard materials and seawater. A longitudinal study of aluminum alloys during friction showed cellular structure development, which may be considered as a synergistic effect since it causes inhomogeneity of a material structure and a decrease in wear rate [37].

Taken together, these results suggest that mechanical properties have an ambiguous effect on friction behavior. Hence, they cannot be accepted as criteria for the tribological properties. Other aspects including material composition, lubricants, environment, carbide decomposition, carbon release, and others were taken into account as well for different friction systems which might be a reason for disputed results. In this regard, the article attempts to investigate the influence of different parameters such as mechanical properties, compositions, and secondary structures formed on the friction surface on tribological parameters of alloys of the same type tested under the same conditions. The most controversial requirements for mechanical properties are imposed on antifriction alloys for journal bearings. Therefore, the research was conducted on new antifriction aluminum alloys developed to replace bronzes [38,39,40]. On the one hand, these materials must have sufficient strength to provide the necessary load-bearing capacity and to resist fatigue failure. On the other hand, relatively high strength and hardness can lead to significant wear of the steel shaft, unreliable running-in, and seizure.

Synergistic effects leading to self-organization reduce wear rate [41,42]. Thus, the fundamental criterion is the ability of an alloy to self-organize through the formation of beneficial secondary structures. They can be found in all the areas of alloy structure: aluminum matrix, hard and soft inclusions. The comprehensive alloying must provide sufficient initial properties of matrix and inclusions as well as compositions variety of the latter. This diversity increases the number of degrees of freedom, types of interactions, and responding reactions of the tribosystem to friction. Importantly, friction is an artificial process, and the reaction of the tribosystem is natural. The natural reaction is aimed at reducing the result of external influences, similar to the Le Chatelier–Brown principle, and proceeds through the formation of protecting films on the friction surface. Their formation involves all the subjects of a tribosystem namely rubbing bodies, lubricants, and the environment [31,41,43].

Since the self-organization processes take place on the friction surface of alloys of the same type tested under the same conditions and the relationship of secondary structures with wear rate will be analyzed. An attempt is made to show that the properties and compositions of alloys are not an unambiguous indicator of wear resistance. Changes in the composition of the friction surface (secondary structures) can be such an indicator. Alloying elements involved in the construction of secondary structures to refine alloying will be identified.

## 2. Materials and Methods

**Figure 1:**
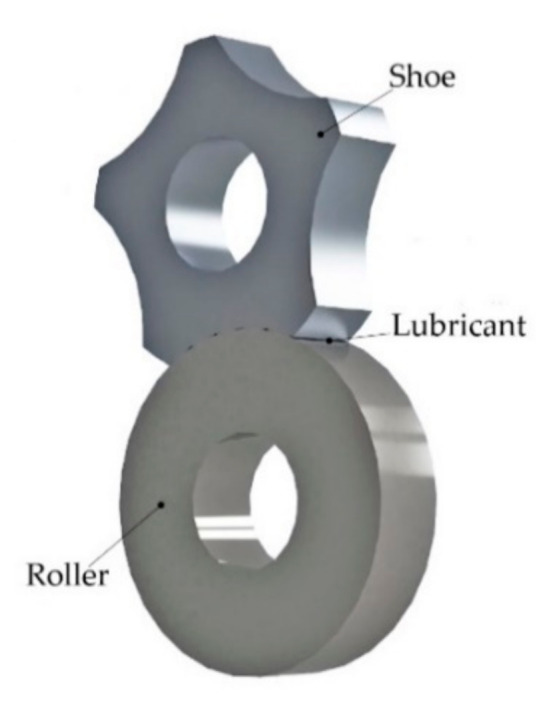
Tribological test scheme.

The tribological tests were carried out based on the shoe-and-roller scheme. The shoe was made of antifriction aluminum alloys while the roller was made of SNC28 chromium-nickel steel (Hakuro Group, Kasai, Japan). The hardness of steel was 250 HB. The radius of the shoe and roller was 20 mm, while their thickness was 10 mm. The steel composition is shown in Table 1. The tribological test scheme is shown in Figure 1. The steel roller rotated at a speed of 500 rpm. The tests were carried out in the API CB oil [44]. The wear tests were conducted after a run-in process of antifriction material and steel counterface. Running-in was carried out until the same contact area for all shoes was reached—200 mm^2^. To estimate wear resistivity, mass loss after tribological tests was measured. The procedure was repeated three times for each material. Different shoes were used for each procedure. The duration of each wear test was 40 h. Details of the test procedures are described in [45].

Chemical compositions of antifriction aluminum alloys was studied by emission spectrometer Spectrolab-S (Spectro Analytical Instruments GmbH, Kleve, Germany). Table 2 provides the chemical composition of the experimental antifriction aluminum alloys.

For the manufacture of alloys the aluminum melt was heated up to a temperature of 800–840 °C, then the Al-Si, Al-Cu Al-Mg additives were introduced into the melt, at a temperature of 760–780 °C, the Sn-Pb-Zn ligature was added into the melt. At a temperature of 740 °C, the melt was poured into a steel mold. Before casting, a modifier-degasser containing titanium was introduced into the melt. The ingot was heat-treated at a temperature of 250 °C for 3 h.

Cast aluminum cylindrical billets were milled to form surfaces for tribological tests (Figure 1). Both the steel rollers and aluminum specimens were polished to Ra = 0.8 μm. The roughness of the samples was studied on a Hommelwerke T8000 profilograph-profilometer (Hommelwerke GmbH, VS-Schwenningen, Germany) with a TKU 300/600 probe with a sensitivity of 40 nm and a step resolution of 1 μm.

In this work, mechanical properties such as hardness (HB), tensile strength (MPa), impact strength KCU (kJ/m^2^) and relative extension (%) were studied. The method for determining the mechanical properties is described in [39,44].

EDX analysis of initial surfaces before and after friction was carried out on a scanning electron microscope (SEM) “Tescan Vega 3” (Czech Republic) equipped with an Oxford Instruments (UK) energy-dispersive analysis module.

To confirm the composition and identify the binding energies of the elements, the samples of aluminum alloys were studied by X-ray photoelectron spectroscopy (XPS) using Thermo Scientific K-alpha (Thermo Fisher Scientific, East Grinstead, UK) equipment with an Al Kα source after preliminary ion etching to a depth of 100 nm. The specimens prior to EDX and XPS analysis were cleaned in an ultrasonic bath with chemically pure acetone.

## 3. Results and Discussion

Table 3, Table 4, Table 5, Table 6 and Table 7 show the research results. Table 3 presents the mechanical properties of the experimental antifriction aluminum alloys.

Table 4 compares the tribological characteristics of the experimental antifriction aluminum alloys.

Table 5 and Table 6 show the results of the EDX-analysis of testing surfaces of the experimental antifriction aluminum alloys before and after friction, respectively.

It is apparent from Table 4 that the AO-5.8 alloy is the most wear-resistant although its hardness, tensile strength, and toughness are rather low. In contrast, the AO-9.6 alloy, having the second-highest hardness and tensile strength, exhibited the almost poorest wear rate and the most intensive wear of the steel counterface. In fact, according to the Table 2, Table 3 and Table 4, the first four alloys containing maximum tin content (8.7–11.0%) outperform the second group of alloys containing lower tin (5.4–7.6%) in mechanical properties while their wear resistivity is worse. The only exception is the AO-9.8 alloy. Alloys with increased content of soft structural components and with increased strength have a higher wear rate compared to alloys with reduced content of soft structural components and with reduced strength properties. This outcome is contrary to previous concepts which have suggested that increasing the content of soft structural components is beneficial. In turn, the AO-9.8 alloy has relatively high hardness and strength while its wear rate is rather low. It can be concluded from comparing Table 2 and Table 4 that low wear rates are common for alloys with a higher content of magnesium and silicon. From this point of view, the AO-7.6 alloy comes as an exception since it has insignificant magnesium content. Overall, no evidence was found for establishing decisive correlations between mechanical properties and wear rates. However, it can thus be suggested that doping alloys by more than 6% tin is excessive, and adding magnesium and silicon is preferred to improve wear resistivity.

Na, Cl, Ca, K, S, and P could fall on the friction surface from oil.

No significant correlation was found for mechanical properties, alloys composition, and seizure loads (Table 2, Table 3 and Table 4). The alloy AO-11 having the highest tin content showed the lowest seizure load, while minimum tin content leads to the highest seizure load (AO-5.4 alloy). Neither was found an influence of other alloying elements on seizure load but magnesium. Only alloys without (AO-11) or with low content (AO-7.6) of magnesium demonstrated poor seizure loads. Favorable values of seizure loads were registered for alloys with magnesium content over 0.3%. From the data, it can be seen that the best seizure performance was achieved by the most magnesium-doped alloy AO-5.4 (Table 2 and Table 4).

To link changes in composition with wear rates friction surface was analyzed and compared to its initial state (Table 5 and Table 6).

Recalculated values of the tin content in secondary structures were calculated based on Table 5 and Table 6. To do this, the content of carbon and oxygen was subtracted and the acquired value was taken as 100%. The percent of the rest elements was recalculated according to the obtained value. According to the results, the most wear-resistant alloys (AO-7.6, AO-6.4, AO-5.8, and AO-5.4) have increased recalculated tin content varying from 3.8% to 6.4%. In contrast, recalculated tin values of the alloys with poor wear rates (AO-11, AO-9.6, and AO-8.7) were ranged from 3.1 to 3.5%.

The alloys AO-9.8, AO-7.6, AO-6.4, AO-5.8, and AO-5.4 with low wear rates were observed to have increased recalculated values of silicon ranged from 1.0% to 13.0%. Two alloys (AO-11, AO-9.6) with high wear rates have lower recalculated values of silicon (0.3% and 0.4%). The AO-8.7 alloy is an exception since it showed the maximum wear-resistance with recalculated silicon content (4.1%).

High magnesium recalculated values from 0.45% to 1.05% are common for the alloys with low wear rates (AO-9.8, AO-7.6, AO-6.4, AO-5.8, AO-5.4). The rest alloys contain only 0.15–0.30% of magnesium in recalculated values. Being the most wear-resistant, the AO-5.8 alloy contains 1.5% Mg in its secondary structures or 1.9% Mg in recalculated values. The second best alloy AO-5.4 and AO-6.4 contain 1.7% and 1.4% of magnesium respectively which corresponds to 1.8% Mg after recalculating. In general, therefore, it seems that 1.5% of magnesium content is optimal. Magnesium in alloys participates in the formation of a solid solution based on aluminum. The release of magnesium from a solid solution is a non-spontaneous phase transition accompanied by negative entropy production. According to the equilibrium diagram to Al-Mg solubility of magnesium in aluminum-based solid solution at 100 °C is about 3%wt and increased with heating [46]. In this study, the content magnesium did not exceed 1.5%wt. Therefore, precipitation of magnesium from solid solution is in contradiction with the equilibrium diagram and considered to be a non-spontaneous process with negative entropy production. Thus, it is possible to infer that this process is a part of dissipative structures occurring during friction. Alloys (AO-11, AO-9.6, AO-9.8) with higher wear rates showed changes in magnesium, content on the surface from -0.08% to 0.19%wt, while for more wear-resistant alloys this range was from 0.3% to 0.7%wt. Hence, it could conceivably be suggested, that precipitation of magnesium is one of the key processes for low wear rate performance.

At the same time, the AO-5.8 alloy contains significantly more oxygen and carbon on its surface (by 1.5–2.5 times) than the other alloys. Despite that, the recalculated values of tin, lead, and magnesium in this alloy are greater. Most of the carbon, oxygen and all of the sulfur on the friction surface is contained in the solid residues of the lubricant. Increased content of O, C, S indicates an increased amount of hardened lubricant residues on the surface. The increased content of carbon and oxygen on the friction surface of the AO-5.8 alloy may be associated with severe conditions in the friction zone. These conditions caused intense self-organization during friction of the AO-5.8 alloy.

Table 7 presents the results of XPS analysis of the elemental composition of secondary structures on the friction surface of the AO-5.8 and AO-8.7 alloys with the lowest and highest wear rates respectively. Only the surface layer up to 10 nm is involved in analysis within this method. Binding energies and other related results are provided in [45].

The content of tin and magnesium in the secondary structures of the AO-5.8 alloy is almost an order of magnitude higher than that of the AO-8.7 alloy. The lead content is 1.7 times higher, which, brought together, confirms the abovementioned findings. It is interesting to note from Table 7 that the AO-8.7 alloy has 2 times more iron on its surface compared to the AO-5.8 alloy. The same comparison based on Table 6 shows that the AO-8.7 alloy contains 4–8 times more iron than the other alloys. The observed increase of iron could be attributed to the more intensive seizure of the AO-8.7 alloy with the steel counterpart during friction.

The present study raises the impossibility of the preliminary establishment of an unambiguous criterion for the minimum wear rate in terms of mechanical properties and alloy compositions. To a certain degree, it may be formulated based on the results of secondary structures analysis, however, tribological tests are required. The initiation of self-organization and the formation of dissipative structures during friction, leading to a decrease in the intensity of wear, is a probabilistic process since the conditions for self-organization are necessary but not sufficient [47].

The mechanisms of self-organization of these alloys are discussed in [44,45]. The self-organization mechanism during friction of the AO-5.8 alloy is characterized by the mass transfer of magnesium from the alloy to the friction surface. This leads to an increase in the concentration of magnesium in the surface layers and the precipitation of magnesium from the solid solution based on aluminum. Such mass transfer and phase transformation contradicts equilibrium phase diagrams and the irreversible diffusion process. Such a non-spontaneous process is accompanied by a negative entropy production and, according to [36,41], leads to a decrease in the wear rate. In alloy AO-8.7, in the absence of magnesium, zinc exhibits similar behavior. Apparently, the absolute value of the negative entropy production of zinc mass transfer is less than the absolute value of the negative entropy production of magnesium mass transfer. Therefore, the mass transfer of zinc decreases the wear rate to a lesser extent in comparison with the mass transfer of magnesium. Such non-spontaneous processes consume a significant part of the friction energy, which without going through such processes would be spent on wear.

However, self-organization is a probabilistic process; under the same conditions, it may or may not go through [46,47]. Therefore, research is needed to increase the probability of self-organization undergoing friction. In this case, to increase the probability of self-organization, it is necessary to reduce the activation energy of magnesium diffusion in aluminum.

## 4. Conclusions

Possible relations of tribological characteristics of aluminum antifriction alloys with their compositions and mechanical properties are analyzed. No unambiguous relationship was found between the mechanical properties of alloys and their wear resistance. The content of the main alloying element, tin, varied from 5.4 to 11% Sn. It is found that tribologically, it doping more than 6% Sn is excessive. No straightforward relationship was found between the content of alloying elements and the seizure load except for magnesium. Alloys containing more than 0.3% Mg have the highest seizure load values. The lowest seizure load was observed for the alloys either without or with the minimum magnesium content. The alloy with the highest magnesium content showed the maximum seizure load.

The study also showed that the alloys with low wear rates are characterized by increased magnesium, tin, and lead content in their secondary structures. In turn, an order of magnitude lower content of tin and magnesium was found in secondary structures of the alloys with increased wear rates.

The present study raises the impossibility of the preliminary establishment of an unambiguous criterion for the minimum wear rate in terms of mechanical properties and alloy compositions.

Specification of alloying of alloys to reduce their wear rate is possible on the basis of a study of secondary structures.

## Figures and Tables

**Table 1:** Steel composition.

Main Components, %wt							Additives, %wt				
**Ni**	**Cr**	**Mn**	**Si**	**C**	**M**	**Fe**	**Cu**	**S**	**Al**	**V**	**Nb**
2.81	0.681	0.625	0.295	0.35	0.477	94.42	0.087	0.011	0.018	0.185	0.013

**Table 2:** Chemical compositions of antifriction aluminum alloys.

Alloy	Content of Elements, %wt							
	Sn	Pb	Cu	Zn	Mg	Si	Ti	Al
AO 11	11.0	2.6	3.9	2.6	–	0.1	0.01	79.8
AO 9.8	9.8	2.5	4.5	2.4	1.2	0.6	0.03	79.0
AO 9.6	9.6	3.2	4.9	4.4	0.3	0.1	0.02	77.5
AO 8.7	8.7	3.2	3.4	2.9	0.4	0.5	0.03	80.9
AO 7.6	7.6	3.3	4.0	0.5	0.07	1.0	0.06	83.5
AO 6.4	6.4	3.0	4.1	1.9	1.4	0.9	0.01	82.3
AO 5.8	5.8	2.7	4.1	2.3	1.5	1.5	0.03	82.1
AO 5.4	5.4	2.6	3.5	2.3	1.7	0.8	0.03	83.7

**Table 3:** Mechanical properties of antifriction aluminum alloys.

Alloy	Hardness, HB	Strength, MPa	Relative Extension, %	Impact Strength KCU, kJ/m^2^
AO-11	59 ± 2	173 ± 4	2.9 ± 0.1	121 ± 5
AO-9.8	74 ± 2	163 ± 3	0.3 ± 0.1	21 ± 5
AO-9.6	63 ± 2	185 ± 4	3.8 ± 0.1	50 ± 5
AO-8.7	60 ± 2	168 ± 3	5.5 ± 0.1	83 ± 5
AO-7.6	50 ± 2	159 ± 3	4.0 ± 0.1	45 ± 5
AO-6.4	55 ± 2	139 ± 3	2.3 ± 0.1	35 ± 5
AO-5.8	55 ± 2	140 ± 3	1.9 ± 0.1	24 ± 5
AO-5.4	53 ± 2	144 ± 3	2.9 ± 0.1	32 ± 5

**Table 4:** Tribological characteristics of antifriction aluminum alloys.

Alloy	Seizure Load, N	Wear of Material, mg	Friction Coefficient	Wear of Steel, mg
AO-11	1650	1.2 ± 0.1	0.020 ± 0.003	0.6 ± 0.1
AO-9.8	2832	0.7 ± 0.1	0.015 ± 0.002	0.7 ± 0.1
AO-9.6	2107	2.0 ± 0.2	0.019 ± 0.003	2.1 ± 0.3
AO-8.7	2407	2.4 ± 0.2	0.022 ± 0.002	0.8 ± 0.1
AO-7.6	1823	0.5 ± 0.1	0.026 ± 0.004	0.8 ± 0.1
AO-6.4	2767	0.9 ± 0.1	0.014 ± 0.002	1.0 ± 0.2
AO-5.8	2330	0.4 ± 0.1	0.018 ± 0.003	0.6 ± 0.1
AO-5.4	2845	0.5 ± 0.1	0.017 ± 0.001	0.7 ± 0.1

**Table 5:** Testing surfaces EDX analysis of initial surface, %wt.

Alloy	Sn	Pb	Cu	Si	Zn	Mg	C	O	Cr	Al
AO-11	6.7	1.2	3.6	0.1	2.4	-	5.7	4.2	-	76.1
AO-9.8	4.2	0.9	3.6	0.7	2.3	0.2	6.3	4.1	0.3	77.4
AO-9.6	5.1	2.1	3.9	0.1	4.0	0.1	5.2	3.9	-	75.6
AO-8.7	4.8	2.2	2.8	0.6	2.6	0.2	6.1	4.7	-	76.0
AO-7.6	4.9	2.0	3.4	1.5	0.8	-	3.8	3.1	-	80.5
AO-6.4	5.0	1.5	3.5	1.3	2.2	0.3	5.4	4.5	-	76.3
AO-5.8	4.2	1.8	3.6	2.2	2.2	0.3	6.2	4.3	-	75.2
AO-5.4	4.1	1.2	2.9	1.3	2.4	0.5	4.9	4.5	-	78.2

**Table 6:** Compositions of secondary structures on friction surfaces of antifriction aluminum alloys.

Alloy	Content of Elements, %wt																	
	Sn	Pb	Cu	Zn	Mg	Si	Cr	Al	C	O	Na	Cl	Ca	K	S	P	Fe	Mn
AO-11	2.7	0.9	4.7	2.0	0.1	0.3	–	60.2	17.0	11.4	0.1	0.1	0.1	–	–	–	0.3	–
AO-9.8	3.2	0.5	4.5	1.8	0.6	1.3	–	68.0	11.3	8.1	–	–	–	–	0.2	–	0.3	–
AO-9.6	2.2	0.7	3.6	3.4	0.2	0.2	–	56.9	18.1	13.6	0.3	0.2	0.1	0.1	–	–	0.4	0.1
AO-8.7	1.9	0.6	2.8	2.0	0.1	2.5	0.1	50.8	21.9	15.1	0.3	0.1	0.1	0.1	–	–	1.6	–
AO-7.6	3.6	1.3	5.4	0.9	0.3	9.1	–	49.3	17.0	12.3	–	0.1	0.2	–	–	0.1	0.3	–
AO-6.4	3.7	0.6	4.8	1.9	0.8	1.6	–	64.1	12.8	8.9	–	0.1	0.1	–	0.1	0.1	0.4	–
AO-5.8	3.2	2.6	1.9	1.4	0.4	0.5	–	39.7	34.6	15.1	–	–	0.1	–	–	–	0.2	–
AO-5.4	2.8	0.9	3.3	1.8	0.6	2.1	–	59.9	18.6	9.7	–	–	0.1	–	–	–	0.2	–

**Table 7:** Results of XPS analysis of the elemental composition of secondary structures on the friction surface of the AO-5.8 and AO-8.7 alloys.

Alloys	Content of Elements, %wt														
	C	O	Pb	Sn	Al	Mg	Zn	Cu	Fe	Si	S	Na	Cl	Ca	K
AO-5.8	29.96	16.22	15.68	12.44	11.72	4.27	1.08	1.05	0.85	0.59	4.6	0.46	0.51	0.31	0.26
AO-8.7	43.45	23.17	9.02	1.1	11.62	0.54	2.77	3.28	1.68	1.06	0.65	0.63	0.78	0.26	0.23

## Data Availability

The data presented in this study are available on request from the corresponding author. The data are not publicly available because the authors do not wish to publish supplementary materials.
